# Suppression of NOD-like receptor protein 3 inflammasome activation and macrophage M1 polarization by hederagenin contributes to attenuation of sepsis-induced acute lung injury in rats

**DOI:** 10.1080/21655979.2022.2047406

**Published:** 2022-03-10

**Authors:** Lin Wang, Min Zhao

**Affiliations:** Department of Emergency Medicine, Shengjing Hospital of China Medical University, Shenyang, Liaoning, China

**Keywords:** Hederagenin, sepsis-induced acute lung injury, macrophage, NLRP3, NF-κB

## Abstract

Acute lung injury (ALI) is a major leading cause of death in sepsis patients. Hederagenin (HG), derived from *Hedera helix Linné*, has anti-inflammatory effects, while its role in sepsis-induced ALI has not been elucidated. *In vivo*, rats were subjected to cecal ligation and puncture to induce ALI and then treated with HG (12.5, 25, or 50 mg/kg) by gavage. Administration of HG raised survival rate, ameliorated lung injury, and decreased lung wet/dry ratio and inflammatory cell accumulation in bronchoalveloar lavage fluid (BALF) of ALI rats. HG inhibited macrophage polarization toward the M1 phenotype as evidenced by decreased CD86 expression in rat lung tissues. Moreover, HG decreased the secretion of TNF-α, IL-6 and monocyte chemoattractant protein-1 (MCP-1) in BALF and the levels of inducible nitric oxide synthase (iNOS) and cyclooxygenase-2 (COX-2) in lung tissues. *In vitro*, phorbol-12-myristate-13-acetate (PMA)-differentiated THP-1 macrophages were stimulated with 100 ng/mL lipopolysaccharide. HG treatment inhibited M1 macrophage polarization and the production of M1-related pro-inflammatory mediators (IL-6, MCP-1, iNOS, and COX-2). Mechanistically, HG inhibited NLRP3 inflammasome activation and subsequent release of IL-18 and IL-1β, and suppressed NF-κB signaling pathway both *in vivo* and *in vitro*. Notably, HG treatment further emphasized the inhibitory effect of NF-κB inhibitor BAY11-7082 on NLRP3 inflammasome activation and macrophage M1 polarization. Taken together, HG exerts a protective effect against sepsis-induced ALI by reducing the inflammatory response and macrophage M1 polarization, which may involve NF-κB pathway-modulated NLRP3 inflammasome activation.

## Graphical abstract

**Figure:**
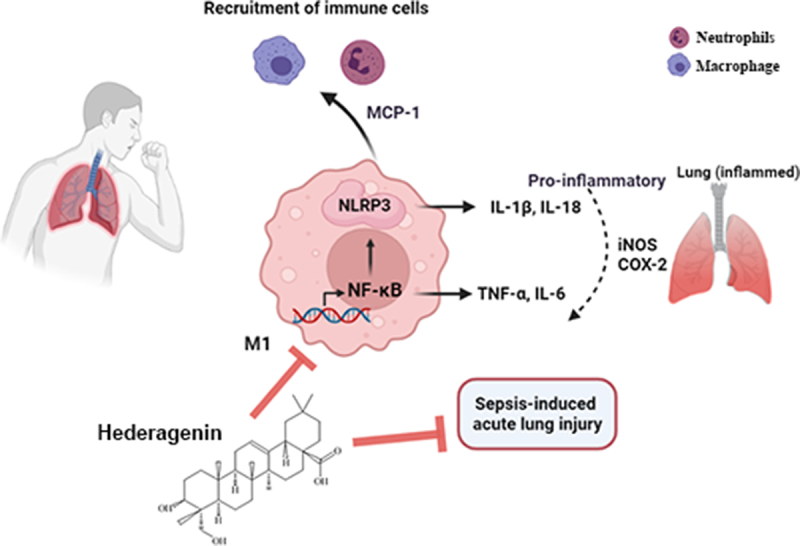

## 1 Introduction

Sepsis is defined as life-threatening organ dysfunction due to the dysregulation of host response to infection. It is a major worldwide health-care problem with high mortality [1,2] [PMID: 21,128,753; 28,101,605]. Almost 30% of patients with sepsis can develop into multi-organ dysfunction syndrome (MODS) [3] [PMID: 28,792,873]. MODS is the most common syndrome induced by sepsis, including acute lung injury (ALI), renal failure, and coagulopathy [4] [PMID: 26,903,338]. Lung is the most vulnerable organ during sepsis. ALI or respiratory distress syndrome (ARDS) develops in about 50% of cases with sepsis [5] [PMID: 19,758,459]. In recent years, the pathogenesis of ALI caused by sepsis has been a matter of great concern. Research showed that ALI is caused by excessive pulmonary inflammatory response and is characterized by respiratory distress with diffuse endothelial and epithelial injury, inflammatory cell infiltration, and pro-inflammatory cytokine release [6] [PMID: 24,745,331]. At the moment, there is no effective clinical treatment for patients with ALI. Antibiotics and supportive measures are the only available treatments with limited effect in reducing mortality [7] [PMID: 30,134,811]. Consequently, exploring the possible mechanisms and developing effective therapeutic strategies are urgent problems to be solved.

The NOD-like receptor protein 3 inflammasome (NLRP3) is a cytoplasmic multi-protein complex that consists of NLRP3, apoptosis-associated speck-like protein (ASC) and pro-caspase-1 [8] [PMID: 20,428,172]. This complex is responsible for the activation of caspase-1 and the production of interleukin (IL)-1β and IL-18 (pro-inflammatory cytokines). Researches have shown that NLRP3 inflammasome plays a critical role in the pathogenesis of inflammatory conditions including sepsis [9] [PMID: 31,741,197]. Inhibition of NLRP3 activation in lipopolysaccharide (LPS)-treated mice has been reported to alleviate the development of ALI [10] [PMID: 27,643,555]. Therefore, NLRP3 inflammasome may be a therapeutic target for ALI. Furthermore, nuclear factor-κB (NF-κB) participates in the pathogenesis of organ injury induced by sepsis [11] [PMID: 9,876,974]. NF-κB, a transcription factor complex, appears to regulate acute inflammation by activating cytokine cascade and the production of pro-inflammatory mediators. In a review of published study, Inhibition of NF-κB increased survival rates of septic mice with LPS attack [12] [PMID: 16,531,564]. NF-κB, as a key mediator of immunity, is essential for NLRP3 inflammasome activation [13] [PMID: 19,570,822]. Studies have reported that disruption of the connection between NF-κB and NLRP3 protected mice from sepsis-induced cardiac damage [14] [PMID: 26,045,547].

Hederagenin (HG) is a pentacyclic triterpenoid compound isolated from *Hedera Helixé (a*lso known as common ivy). Recently, HG has been reported to have anti-cancer [15] [PMID: 19,406,196], anti-inflammatory [16] [PMID: 26,481,049], hyperlipidemia therapy [17] [PMID: 26,557,859], anti-depressant [18] [PMID: 25,471,378] and liver protection [19] [PMID: 28,067,819] effects *in vitro* and *in vivo*. HG could inhibit inflammatory cell infiltration and mast cell degranulation to alleviate LPS-induced inflammatory response in rats [16] [PMID: 26,481,049]. A previous study has indicated that HG decreased the expression of inflammatory cytokines through the deactivation of NF-κB signal pathway [17] [PMID: 26,557,859].

Based on above findings, we hypothesized that HG might play a role in sepsis-induced ALI by regulating inflammatory response and macrophage activation through the NLRP3/NF-κB axis. Hence, we established a cecal ligation and puncture (CLP)-induced ALI model in Sprague-Dawley (SD) rats and treated ALI rats with HG to evaluate its potential therapeutic effect on ALI and explore its mechanism.

## 2 Material and methods

### 2.1 Animals

All animal procedures in this research were performed according to the international guidelines for care and use of laboratory animals as well as approved by the Ethics Committee of Shengjing Hospital of China Medical University.

SD rats (Male, 250–300 g, 8 weeks old) were purchased from Liaoning Changsheng biotechnology co., Ltd. All rats were housed in cages with temperature-controlled (25 ± 1°C) and humidity-controlled condition (50 ± 5%). Food and water were freely accessed. To establish a rat model of sepsis-induced ALI, rats underwent CLP surgery [20] [PMID: 22,059,996]. In short, rats were anesthetized, and an incision was made in the abdominal wall to expose the cecum. A puncture was performed in the distal cecum with 22-G needle for twice, and the cecum was then ligated with 3–0 silk thread. Sham-operated rats only underwent laparotomy without ligation and puncture. Immediately post-procedure, all rats were given 12.5, 25, or 50 mg/kg HG (Aladdin, Shanghai, China) once by gavage [21] [PMID: 32,024,440]. The experiment was terminated at 12 hours post-CLP, and the lung tissues and bronchoalveolar lavage fluid (BALF) were obtained. Meanwhile, 10 rats of each group were selected randomly to observe the survival rate.

### 2.2 Wet/dry (W/D) ratio

The lung tissues in part of rats were collected to weigh wet weight, and then bake it in 80°C oven until the dry weight was constant. The W/D ratio was calculated by dividing the wet weight by the dry weight.

### 2.3 BALF analyses

The left lung was washed three times to collect the BALF. After collection, the BALF were centrifuged and re-suspended in 500 μL phosphate buffered saline (PBS). Total leukocyte was measured using a hemocytometer. Differential cell counts were determined manually by Giemsa staining (Jiancheng Bioengineering Institute, Nanjing, China) of cell pellet smear from BALF [22] [PMID: 30,443,024]. The cells were identified as macrophage, neutrophil and lymphocyte using standard morphological criteria under the microscope (Olympus, Tokyo, Japan).

### 2.4 Cell culture and group

The human monocyte leukemia cell line (THP-1) (iCell Bioscience Inc., Shanghai, China) was maintained in RPMI-1640 culture medium (Solarbio, Beijing, China) containing 10% fetal bovine serum (FBS) at 37°C in an incubator with 5% CO_2_. In experiments, THP-1 monocytes were treated with 100 ng/mL phorbol-12-myristate-13-acetate (PMA) (Aladdin, Shanghai, China) for 24 h to differentiate into macrophage-like cells [23] [PMID: 32,117,982].

The differentiated THP-1 cells were divided into four groups: control, control+HG, LPS and LPS+HG group. The cells were treated with HG (100 μM) for 1 h, followed by 100 ng/mL LPS (Solarbio, Beijing, China) stimulation for 24 h to activate THP-1 macrophages [24] [PMID: 26,481,049].

### 2.5 Histopathological analyses

The lung injury was determined according to the Hematoxylin and Eosin (H&E) staining [25] [PMID: 30,797,018] using related pathological scoring criteria [26] [PMID: 25,887,405]. Rats lung tissues were embedded in paraffin section (5 μM) and stained with hematoxylin (Solarbio, Beijing, China) and eosin (Sangon Biotech, Shanghai, China) in accordance with standard procedures. The light microscopy (Olympus, Tokyo, Japan) was employed for the observation of the degree of lung injury. In addition, the severity of lung tissues were scored according to the presence of exudates, hyperemia/congestion, intra-alveolar hemorrhage/debris, cellular infiltration, and cellular hyperplasia [27] [PMID: 25,887,405] where grade 0 (0% negative), grade 1 (0–33% mild positive), grade 2 (33–66% moderate positive), grade 3 (66–100% severe positive). The p-p65 antibody (diluted 1:100; Novus Biologicals, Littleton, CO, USA) was used for immunohistochemistry (IHC) of phos-p65 in the paraffin embedded lung tissue sections. Images were collected using an optical microscope (Olympus, Tokyo, Japan).

### 2.6 Immunofluorescence assay

Immunofluorescence staining was performed as previously described with minor modification [28] [PMID: 33,472,663]. The lung tissue or THP-1 cell sections were blocked with 1% bovine serum albumin (BSA) (Sangon Biotech, Shanghai, China) for 15 min and incubated with anti-CD68 (diluted 1:50; Santa Cruz Biotechnology, Inc., Santa Cruz, CA, USA), anti-CD86 (diluted 1:100; ABclonal Technology, Wuhan, China)), anti-iNOS (diluted 1:100; Affinity Biosciences, Changzhou, China), anti-NLRP3 (diluted 1:100; ABclonal Technology, Wuhan, China), anti-ASC (diluted 1:50; Santa Cruz Biotechnology, Inc., Santa Cruz, CA, USA) and anti-p65 (diluted 1:400; CST, Danvers, MA, USA) overnight. Subsequently, the slices were incubated with corresponding secondary antibodies (Cy3: goat-anti rabbit, diluted 1:200, Invitrogen, Carlsbad, CA, USA; FITC: goat-anti-mouse, diluted 1:200, Abcam, Cambridge, UK), followed by counterstaining with 4’, 6-diamidino-2-phenylindole (DAPI) (Aladdin, Shanghai, China). Fluorescence microscope (Olympus, Tokyo, Japan) was used to capture stained images.

### 2.7 Western blot

The experimental operation referred to the method of Barczak *et al*. [29] [PMID: 29,654,294]. Total or nuclear proteins from lung tissue and THP-1 cell lysates were prepared using radio-immunoprecipitation assay (RIPA) lysis (Solarbio, Beijing, China) solution or nuclear protein extraction kit (Solarbio, Beijing, China). A total of 10–20 μg protein were separated in sodium dodecyl sulfate polyacrylamide gel electrophoresis (SDS-PAGE) (Solarbio, Beijing, China) and transferred to polyvinylidene fluoride (PVDF) (Millipore, Billerica, MA, USA) membrane for 90 min. Then, the membranes were blocked with 5% nonfat dry milk or BSA buffer for 60 min and incubated with primary antibodies: anti-p-IκBα, anti-IκBα, iNOS (diluted 1:500; Affinity Biosciences, Changzhou, China), anti-p-p65 (diluted 1:500; Novus Biologicals, Littleton, CO, USA), anti-p65 (diluted 1:500; CST, Danvers, MA, USA), anti-COX-2 (diluted 1:1000), anti-NLRP3 (diluted 1:500; ABclonal Technology, Wuhan, China), or anti-caspase-1 (diluted 1:1000; Beijing Biosynthesis Biotechnology, Beijing, China) overnight at 4°C with gentle agitation. After incubation with corresponding horseradish peroxidase (HRP)-conjugated secondary antibodies (goat anti-rabbit IgG or goat anti-mouse IgG) (diluted 1:3000; Solarbio, Beijing, China) for 60 min, the protein blots were visualized by electrochemiluminescence (ECL) kit on Gel imaging system (Beijing Liuyi Biotechnology Co., Ltd., Beijing, China). The protein expression was normalized to GAPDH (diluted 1:5000; Solarbio, Beijing, China) or Histone H3 (diluted 1:1000; Affinity Biosciences, Changzhou, China).

### 2.8 Reverse transcription-quantitative PCR (RT-qPCR)

RT-qPCR protocols follow previous reference [30] [PMID: 30,391,287]. TRIpure (BioTeke Bio., Beijing, China) method was adopted to extract total RNA from rat lung tissues or THP-1 cells. The extracted-RNA was reversely transcribed into cDNA by BeyoRT II M-MLV reverse transcriptase (Beyotime Biotech Co., Ltd., Shanghai, China). RT-qPCR was conducted with 2× Taq PCR MasterMix (Solarbio, Beijing, China) and SYBR green (Solarbio, Beijing, China) on a real-time PCR system (Bioneer Corporation, Daejeon, Korea). The data were analyzed with the 2^−ΔΔCt^ method. GAPDH was used as internal control. The gene primers were presented as follows: COX-2: F: 5’-GAA CAC GGA CTT GCT CAC TT-3’, R: 5’-ACG ATG TGT AAG GTT TCA GG-3’; iNOS: F: 5’-TTG GAG CGA GTT GTG GAT TG, R: 5’-GTG AGG GCT TGC CTG AGT GA-3’.

### 2.9 Flow cytometry

The treated THP-1 cells were trypsinized and centrifuged, and then re-suspended in flow cytometry staining buffer. For surface marker staining, cells were incubated with anti-CD86 (MultiSciences Biotech., Co., Ltd., Hangzhou, China) for 30 min in dark area at 4°C [31] [PMID: 31,088,266]. Flow Cytometry was performed on NovoCyte (Agilent Technologies Inc., Santa Clara, CA, USA).

### 2.10 Enzyme-linked immunosorbent assay (ELISA)

Tumor necrosis factor-α (TNF-α), monocyte chemoattractant protein-1 (MCP-1), interleukin (IL)-6, IL-1β, and IL-18 concentrations in BALF, lung tissues, or cell supernatant of THP-1 cells were measured by corresponding ELISA kit [32] [PMID: 34,464,887] (MultiSciences Biotech, Co., Ltd., Hangzhou, China; Wuhan Fine Biotech Co., Ltd., Wuhan, China). All procedures were conformed to the related manufacturer’s printed instructions.

### 2.11 Biochemical analysis

Myeloperoxidase (MPO) and superoxide dismutase (SOD) activities, and malondialdehyde (MDA) and glutathione (GSH) contents were detected by commercially available kit (Jiancheng Bioengineering Institute, Nanjing, China).

### 2.12 Statistic analysis

All data were performed by GraphPad Prism 8.0. Survival rate data were analyzed by the Kaplan–Meier curve, and the log-rank statistical test was applied to compare the curves. The multiple comparisons of different groups were analyzed by one-way ANOVA. All data were described as mean with standard deviation. *P*-value < 0.05 was considered statistically significant.

## 3 Results

In this study, we investigated the role of HG in sepsis-induced ALI using a rat model of sepsis-induced ALI. We hypothesized that HG might have therapeutic effects on sepsis-induced ALI. We further explored the molecular regulatory mechanisms of HG in PMA-differentiated THP-1 macrophages. Finally, we confirmed HG exerts a protective effect against sepsis-induced ALI by inhibiting NF-κB pathway-modulated NLRP3 inflammasome activation.

### 3.1 Hederagenin increases survival rate and attenuates lung injury in rats with sepsis-induced acute lung injury

**Figure 1.**
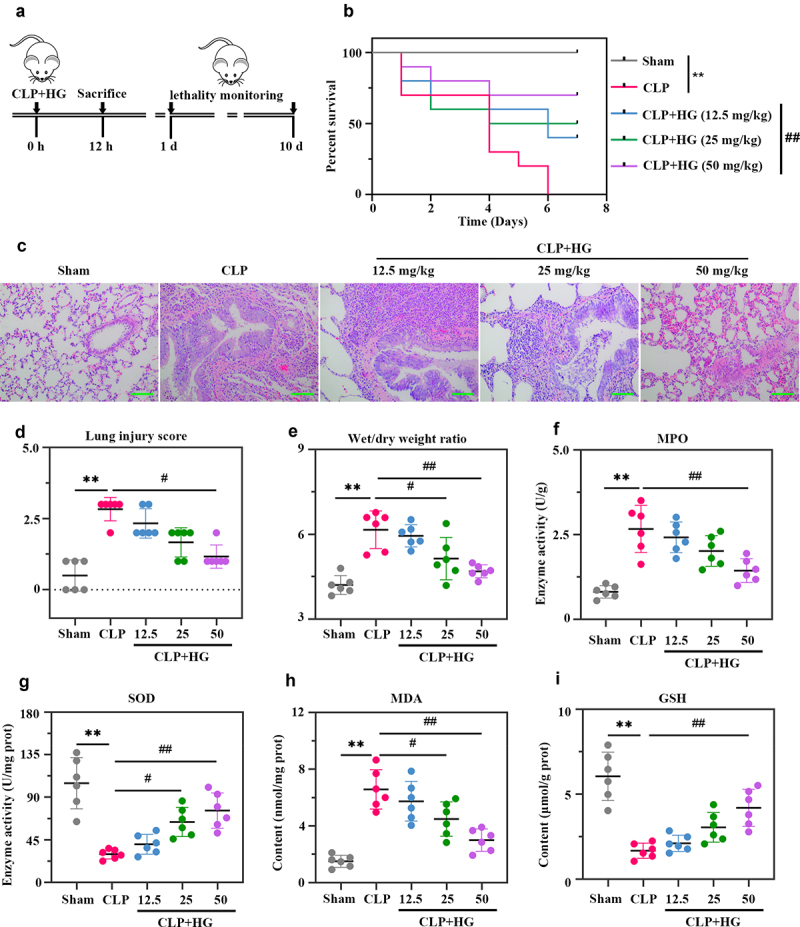
**Hederagenin increases survival rate and attenuates lung injury in rats with sepsis-induced acute lung injury**. (a) Schematic design of the experimental procedure. Rats underwent sham or cecal ligation and puncture (CLP) operation on day at 0. After CLP surgery, rats were immediately given different dosage of hederagenin, gavage administration. Twelve hours after operation, 10 rats in each group were randomly selected to observe the survival rate (10 days or until the model group died); the other part of rats were sacrificed to collect broncho alveolar lavage fluid (BALF) and lung tissues. (b) The survival rate of rats was recorded until the model group died. Results were expressed as percent survival, n = 10. (c) Lung tissue sections stained with hematoxylin and eosin. (Original magnification ×200, scale bar 100 μm). (d) Semiquantitative analysis of lung tissue by lung injury score. (e) The pulmonary edema was measured by wet to dry weight ratio. (f-g) Myeloperoxidase (MPO) (f) and superoxide dismutase (SOD) (g) activities in lung tissue. (h-i) Malondialdehyde (MDA) (h) and glutathione (GSH) (i) content in lung tissue. Results were presented as mean ± standard deviation, n = 6. **P < 0.01 versus Sham group; ^#^*P* < 0.05 versus CLP group; ^##^*P* < 0.01 versus CLP group.

In this study, we investigated the effect of HG on sepsis-induced ALI. The results showed that the rat survival rate was remarkable decreased after CLP surgery. Administration of 50 mg/kg HG after CLP significantly increased the survival rate of ALI rats (Figure 1(b)). As shown in the histological images of lung tissue, sham-operated rats presented normal pulmonary alveoli structure, while operated-rats presented thickened alveoli septum, infiltration of inflammatory cells, alveolar damage and edema (Figure 1(c)). HG treatment alleviated CLP-induced morphological changes in lung tissue (Figure 1(c-d)). The results of Figure 1(e) indicated that the W/D ratio was markedly increased in the CLP group, while it was substantially decreased with the treatment of HG. Furthermore, HG treatment effectively reduced the increase in MPO activity and MDA content caused by CLP (Figure 1(f, h)), and it also increased CLP-induced SOD and GSH depletion (Figure 1(g, i)). These findings suggested that HG attenuated CLP-induced ALI in rats.

### 3.2 Hederagenin suppresses M1 macrophage activation and inflammation response in septic lung tissue

**Figure 2.**
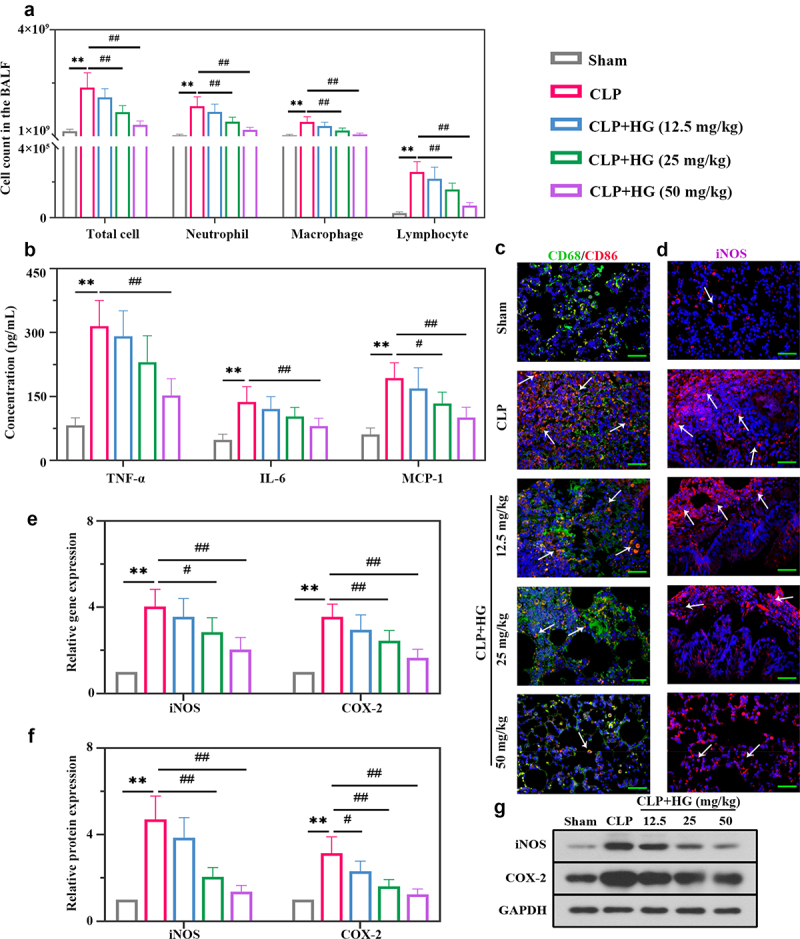
**Hederagenin suppresses M1 macrophage activation and inflammation response in septic lung tissue**. (a) Total cell, neutrophil, macrophage and lymphocyte counts in BALF. (b) Inflammatory factor (tumor necrosis factor-α (TNF-α), interleukin 6 (IL-6) and monocyte chemoattractant protein-1 (MCP-1) levels in BALF. (c) Dual immunofluorescence staining of CD68 (green) and CD86 (red) in the different groups after CLP. (d) Immunofluorescence staining of inducible nitric oxide synthase (iNOS) in the different groups after CLP (Original magnification ×400, scale bar 50 μm). (e-f) iNOS and cyclooxygenase-2 (COX-2) gene (e) and protein (f) expression in lung tissue were detected by quantitative reverse transcription polymerase chain reaction (RT-qPCR) and western blot. (g) Representative images show the expression levels of iNOS and COX-2 by western Blot analysis with GAPDH as a loading control. Results were presented as mean ± standard deviation, n = 6. **P < 0.01 versus Sham group; ^#^*P* < 0.05 versus CLP group; ^##^*P* < 0.01 versus CLP group.

We subsequently analyzed inflammatory cell infiltration in BALF. As expected, ALI induced a significant increase in total inflammatory cells, neutrophils, macrophages, and lymphocytes, but evidently ameliorated by the treatment with HG on dosage of 25 and 50 mg/kg (Figure 2(a)). We detected the levels of pro-inflammatory and chemotactic cytokines in BALF. ELISA analysis revealed elevated levels of TNF-α, IL-6, and MCP-1, and these changes were inhibited by HG treatment (Figure 2(b)). Macrophages are important effectors for host defense against external stimuli, and their activation promotes the lung inflammation involved in ALI [33] [PMID: 30,173,054]. Thus, we investigated the expression of macrophage surface marker CD68 and CD86 to evaluate the role of HG on macrophage activation. Immunofluorescence staining showed an increased number of CD86-positive macrophages and an increased ratio of CD86/CD68 (CD86: M1-specific surface marker; CD68: M0-specific surface marker), indicating a trend of M1-induced macrophage polarization in the lung tissues of ALI rats. However, M1 macrophage polarization was inhibited following the treatment with HG (Figure 2(c)). Inducible nitric oxide synthase (iNOS) and cyclooxygenase-2 (COX-2) are vital inflammatory factors that contribute to macrophage M1 polarization. Consistent results of iNOS were observed using immunofluorescence (Figure 2(d)). After challenge with CLP, HG administration resulted in an observable decrease in iNOS and COX-2 expression at the mRNA and protein levels (Figure 2(e-g)). Collectively, these data indicated that HG administration inhibited CLP-induced M1 macrophage polarization and pulmonary inflammation in ALI rats.

### 3.3 Hederagenin inhibits the activation of NLRP3 inflammasome in septic lung tissue

**Figure 3.**
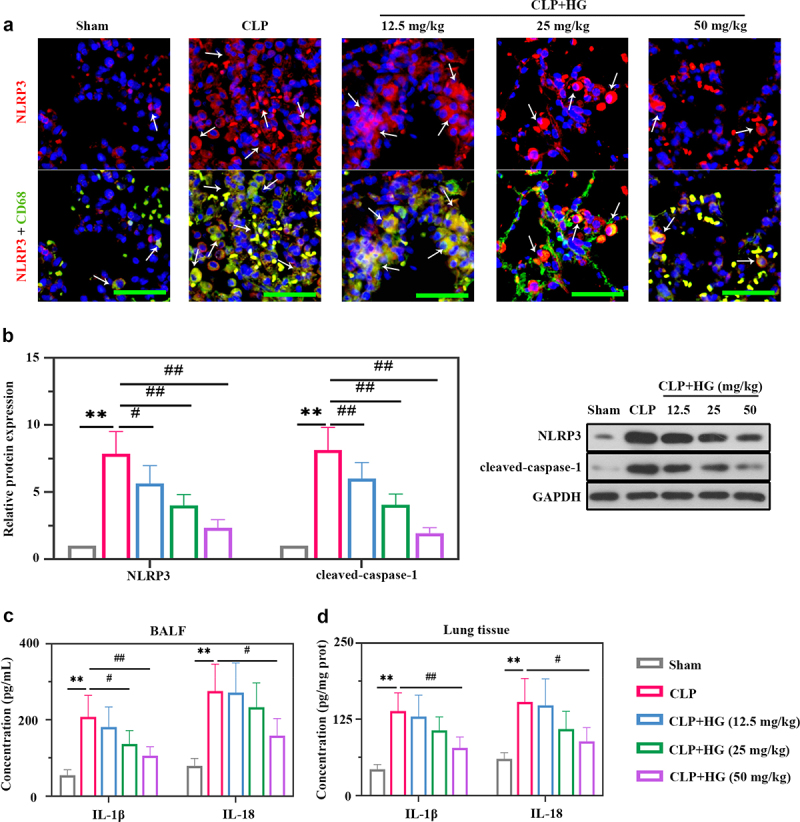
**Hederagenin inhibits the activation of NLRP3 inflammasome in septic lung tissue**. (a) Immunofluorescence staining of NOD-like receptor family pyrin domain containing 3 (NLRP3) and dual immunofluorescence staining of NLRP3 (red) and CD68 (green) in the different groups after CLP (Original magnification ×400, scale bar 50 μm). White arrows indicated positive staining. (b) Protein expression levels of NLRP3 and cleaved-caspase-1 in lung tissue were measured by western blot. Quantitative analysis of the blots was normalized to GAPDH. (c-d) Inflammatory cytokine levels were detected in the BALF (c) and lung samples (d). Results were presented as mean ± standard deviation, n = 6. **P < 0.01 versus Sham group; ^#^*P* < 0.05 versus CLP group; ^##^*P* < 0.01 versus CLP group.

The role of NLRP3 in ALI has been well identified previously [34] [PMID: 33,252,860]. We performed dual immunofluorescence staining for NLRP3 and CD68 to explore the expression of NLRP3 and its location. The CD68 staining was co-localized with NLRP3 indicating that macrophages expressed NLRP3 (Figure 3(a)). Moreover, compare with normal tissues, the septic lung tissues contained more CD68-positive cells and higher NLRP3 expression (Figure 3(a)). The degree was decreased after HG treatment (Figure 3(a)), suggesting the inhibitory of HG on NLRP3 expression in macrophages by HG. In our study, we also detected the expression of NLRP3 and caspase-1 by Western blot. Elevated expression levels of NLRP3 and cleaved caspase-1 were observed in septic lung tissues, indicating the activation of NLRP3 inflammasome (Figure 3(b)). Whereas HG treatment conspicuously down-regulated these inflammasome proteins expression (Figure 3(b)). In addition, the levels of IL-1β and IL-18 were increased in BALF and lung tissues of CLP-treated rats, while HG effectively reversed these changes (Figure 3(c-d)).

### 3.4 Hederagenin inhibits NF-κB signaling pathway in septic lung tissue

**Figure 4.**
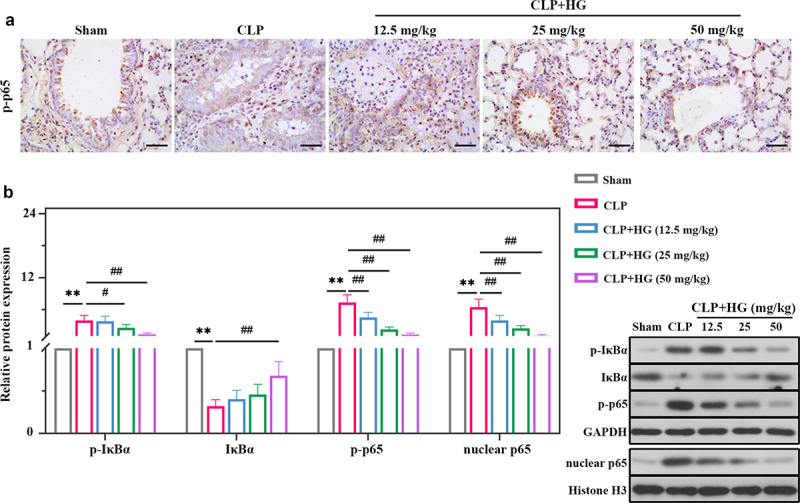
**Hederagenin inhibits NF-κB signaling pathway in septic lung tissue**. (a) Immunohistochemistry staining of p-p65 in the different groups after CLP (Original magnification ×400, scale bar 50 μm). (b) Protein expression levels of p-IκBα, IκBα, p-p65 and nuclear p65 in lung tissue were measured by western blot. Quantitative analysis of the blots was normalized to GAPDH or Histone H3. Results were presented as mean ± standard deviation, n = 6. **P < 0.01 versus Sham group; ^#^*P* < 0.05 versus CLP group; ^##^*P* < 0.01 versus CLP group.

We further explored the role of HG in regulation of NF-κB signaling in lung tissue. As shown in Figure 4(b), the protein expression levels of p-IκBα, p-p65, and nuclear p65 were significantly higher in the CLP group compared with the sham-operated group, while IκBα expression was decreased. In contrast, decreased expression of p-IκBα, p-p65, and nuclear p65, and increased expression of IκBα were observed in the presence of HG (Figure 4(b)). In coincidence with Western blot results, immunohistochemistry staining exhibited that CLP promoted the expression of p-p65, which was significantly reduced after HG treatment (Figure 4(a)).

### 3.5 Effects of hederagenin on LPS-induced macrophages

**Figure 5.**
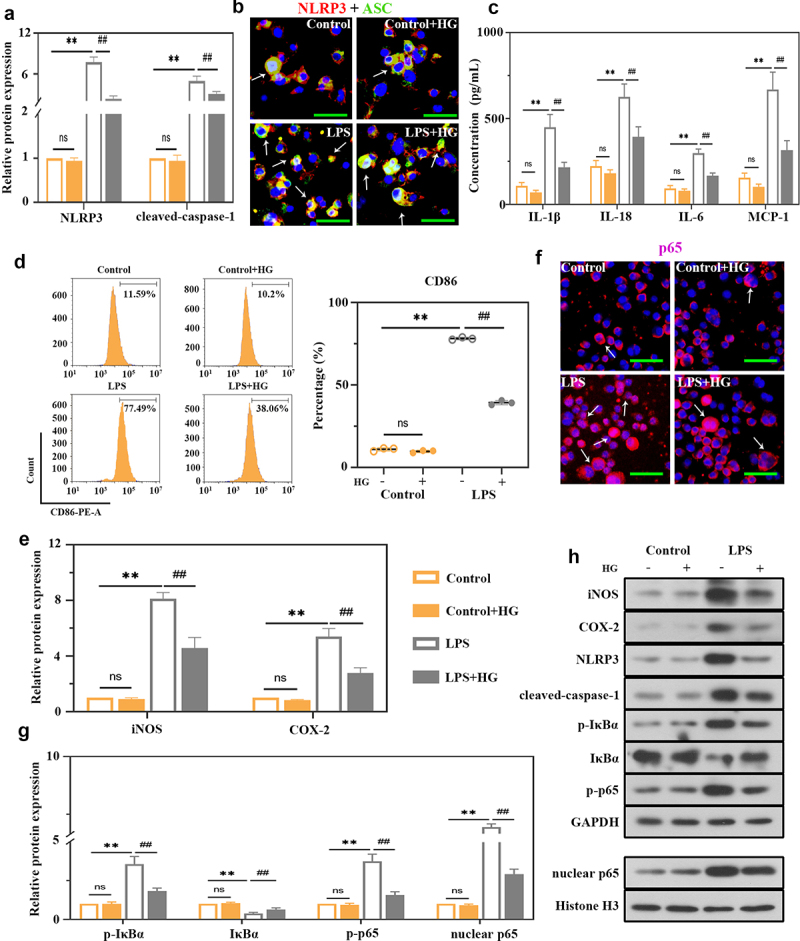
**Effects of hederagenin on LPS-induced macrophages**. THP-1 cells were treated with 100 ng/mL phorbol-12-myristate-13 acetate (PMA) for 24 h to differentiate into macrophage-like cells. Then, the cells were co-cultured with 100 μM hederagenin, followed by 100 ng/mL LPS stimulation for 24 h to activate THP-1 macrophages. (a) Protein expression levels of NLRP3 and cleaved-caspase-1 were measured by western blot after macrophages were treated as indicated. (b) Dual immunofluorescence staining of NLRP3 (red) and ASC (green). (Original magnification ×400, scale bar 50 μm). (c) Inflammatory cytokine levels (IL-1β, IL-18, IL-6 and MCP-1) were detected in macrophages. (d) Flow cytometry was performed to analyze the expression of CD86 on macrophages. (e) Protein expression levels of iNOS and COX-2 in macrophages were measured by western blot. (f) Immunofluorescence staining of p65. (Original magnification ×400, scale bar 50 μm). (g) Protein expression levels of p-IκBα, IκBα, p-p65 and nuclear p65 in macrophages were measured by western blot. (h) Representative images show the expression levels of iNOS, COX-2, NLRP3, cleaved-caspase-1, p-IκBα, IκBα, p-p65 and nuclear p65 by western Blot analysis with GAPDH or Histone H3 as a loading control. Results were presented as mean ± standard deviation, n = 3. No significant (ns), P > 0.05 versus Control group. **P < 0.01 versus Control group; ##P < 0.01 versus LPS group.

To explore the underlying mechanism of HG in macrophages, *in vitro* studies were performed using the THP-1 cell line. Stimulation of THP-1-derived macrophages with LPS induced a significant increase in NLRP3 and cleaved caspase-1 expression (Figure 5(a)) and NLRP3 inflammasome assembly (Figure 5(b)), indicating the activation of NLRP3 inflammasome. Meanwhile, the levels of IL-1β, IL-18, IL-6 and MCP-1 presented the same trends (Figure 5(c)). HG treatment inhibited these alterations in LPS-treated cells (Figure 5(a-c)). As shown in Figure 5(d), the percentage of CD86-expressing cells was obviously increased in LPS-stimulated group. In contrast, HG treatment significantly reduced its percentage (Figure 5(d)). The expression levels of iNOS and COX-2 were also decreased in HG-treated cells (Figure 5(e)). Therefore, HG could inhibit LPS-induced M1 macrophage polarization. We then analyzed the NF-κB signaling activation to unveil the potential mechanism of HG. HG prominently reduced p-IκBα, p-p65, and p65 levels induced by LPS, as well as the translocation of p65 from cytoplasm to nucleus (Figure 5(f)), and increased IκBα levels (Figure 5(g-h)).

### 3.6 Hederagenin suppresses LPS-induced NLRP3 inflammasome activation and M1 macrophage polarization via NF-κB signaling pathway

**Figure 6.**
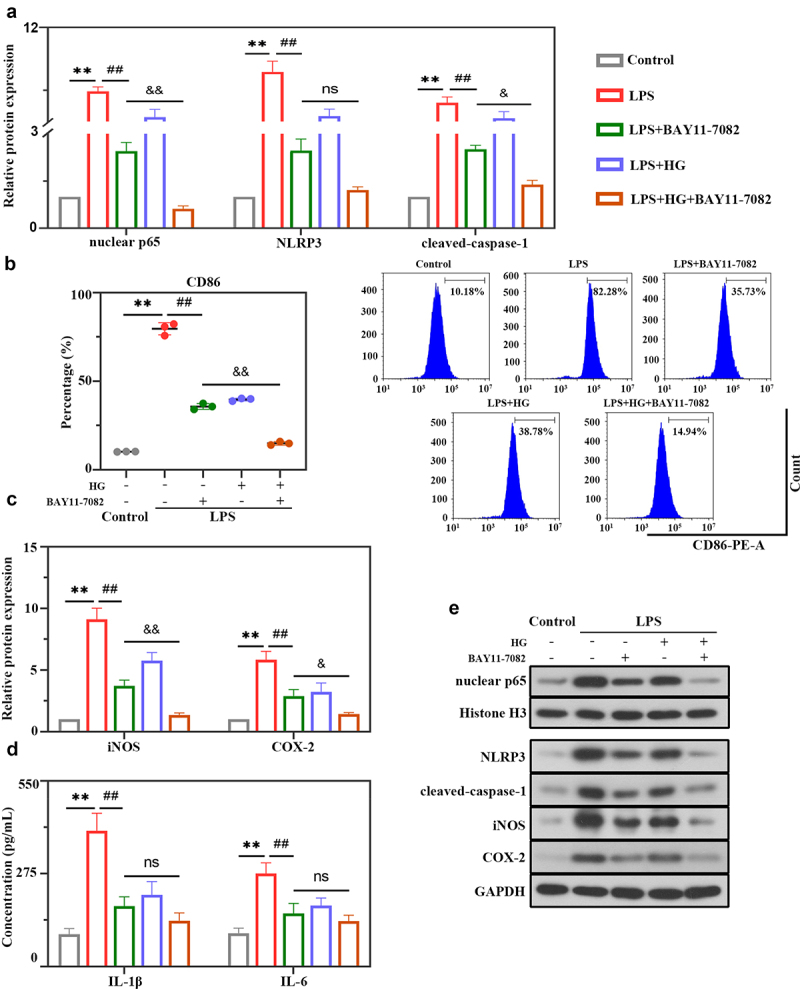
**Hederagenin suppresses LPS-induced NLRP3 inflammasome activation and M1 macrophage polarization via NF-κB signaling pathway**. NF-κB inhibitor was used to further explore the mechanism of hederagenin. The differentiated THP-1 cell were treated with hederagenin and/or BAY11-7082 for 1 h, followed by 100 ng/mL LPS stimulation for 24 h. (a) Protein expression levels of nuclear p65, NLRP3 and cleaved-caspase-1 were measured by western blot after macrophages were treated as indicated. (b) Flow cytometry was performed to analyze the expression of CD86 on macrophages. (c) Protein expression levels of iNOS and COX-2 were measured by western blot. (d) Inflammatory cytokine levels (IL-1β and IL-6) were detected in macrophages. (e) Representative images show the expression levels of nuclear p65, NLRP3, cleaved-caspase-1, iNOS and COX-2 by Western Blot analysis with GAPDH or Histone H3 as a loading control. Results were presented as mean ± standard deviation, n = 3. **P < 0.01 versus Control group; ^##^*P* < 0.01 versus LPS group; No significant (ns), *P* > 0.05 versus LPS+BAY11-7082 group; ^&^*P* < 0.05 versus LPS+BAY11-7082 group; ^&&^*P* < 0.01 versus LPS+BAY11-7082 group.

In next experiments, whether HG is involved in NLRP3 inflammasome activation and macrophage polarization through the NF-κB signaling pathway was further confirmed using the NF-κB inhibitor BAY11-7082. The results showed that BAY11-7082 inhibitor or HG dramatically inhibited LPS-induced expression of nuclear p65 and NLRP3 inflammasome molecules (NLRP3 and cleaved-caspase-1) (Figure 6(a)). As Figure 6(b) showed that BAY11-7082 or HG markedly suppressed CD86 levels as determined by flow cytometry analysis (Figure 6(b)) and reduced the expression of iNOS and COX-2 in LPS-stimulated cells (Figure 6(c)). Moreover, we observed an obvious decrease in the levels of pro-inflammatory cytokines IL-1β and IL-6 in BAY11-7082 or HG-treated cells (Figure 6(d)). Importantly, these inhibitory effects were emphasized by the combination treatment of BAY11-7082 and HG, suggesting that HG modulated the activation of NLRP3 inflammasome and macrophage polarization might largely be through the NF-κB signaling pathway.

## 4 Discussion

ALI is a serious disease with high incidence rate and mortality rate, usually accompanied by acute inflammatory reaction [35]. [PMID: 28,315,822]. In our research, we successfully established an ALI rat model by CLP surgery. We found that HG treatment reduced the mortality of ALI rats. Meanwhile, HG decreased the pathological injury score and W/D ratio of lung tissues. Elevated W/D ratio accounted for increased pulmonary permeability and lung edema [36] [PMID: 23,398,155]. All these indicated that HG administration effectively mitigated the lung injury induced by CLP challenge.

The development of ALI involves multiple pathological processes such as increased capillary permeability, extensive neutrophil infiltration, release of inflammatory mediators, and edema [37] [PMID: 25,596,299]. The severity of ALI has been reported to be related to two processes: recruitment of inflammatory cells and up-regulation of pro-inflammatory cytokines [38] [PMID: 29,163,554]. Our results demonstrated that HG inhibited inflammatory cell infiltration in BALF. Additionally, MPO activity is an effective measure of neutrophil infiltration in lung tissues [39] [PMID: 2,158,602]. It was shown that upregulated recruitment and activation of macrophages and neutrophils could lead to organ damage not only through the release of uncontrolled inflammatory mediators but also the formation of oxidative stress. Oxidative stress leads to excessive production of MPO and MDA, and reduces the expression of antioxidant enzymes such as SOD and GSH, resulting in lung damage in experimental sepsis in rats [40] [PMID: 21,782,879]. Moreover, excessive oxidative stress has been shown to play an important role in the pathogenesis of ALI. It usually leads to pulmonary edema and excessive inflammatory cell infiltration [41] [PMID: 22,850,883]. Duan *et al* reported that inhibition of oxidative stress alleviated LPS-induced ALI in rats [42] [PMID: 32,693,689]. The antioxidant effect of HG has been reported previously [43] [PMID: 29,456,786]. In line with previous studies, HG was found to decrease inflammatory cell infiltration and oxidative stress, thus alleviating lung injury in septic rats caused by CLP. These results indicated that HG had protective effect against CLP-induced ALI *in vivo*.

Macrophages are the most abundant immune cells in lung tissues and are essential for the initiation and maintenance of inflammatory response [44] [PMID: 27,667,687]. It has been well documented that during the inflammatory stage of ALI, inflammation is associated with the phenotype and function of macrophage [45] [PMID: 22,566,854]. During pulmonary inflammation, macrophages differentiate into M1 type and release pro-inflammatory factors (TNF-α, IL-6 and MCP-1). M1 macrophage polarization induces pro-inflammatory response and is involved in the pathogenesis of ALI [46] [PMID: 24,508,730]. In our present study, HG significantly inhibited M1 macrophage polarization both *in vivo* and *in vitro*. Meanwhile, HG treatment effectively reduced CLP-induced the secretion of TNF-α, IL-6, and MCP-1, and inhibited iNOS and COX-2 expression in ALI rats and LPS-stimulated THP-1 cells. These inflammatory mediators are closely associated with M1 macrophages and the development of acute and chronic inflammation diseases [47,48] [PMID: 17,893,865; 16,218,459]. The regulatory effect of HG on macrophage polarization is supported by a previous study showed that HG significantly reduced M1 pro-inflammatory microglia and decreased the levels of TNF-α and IL-6 within the infarcted areas to alleviate cerebral ischemia/reperfusion injury [49] [PMID: 32,848,779]. Therefore, HG might attenuate ALI through mitigation of inflammatory response via inhibition of macrophage polarization toward M1 phenotype.

In the pathogenesis of inflammatory lung diseases, the NLRP3 inflammasome and NF-κB pathways are two important signaling pathways involved in the regulation of the macrophage polarization and inflammatory response [50,51] [PMID: 23,994,575; 23,343,326]. Regardless of the mechanism, lung macrophages play an important role in the pathogenesis of lung injury [52] [PMID: 23,672,262]. Plenty of studies have shown that activation of NF-κB and NLRP3 in macrophage was involved in the inflammatory response in ALI by inducing macrophage M1 polarization [53,54] [PMID: 31,734,560; 32,291,445]. We found that a significant decrease in the percentage of M1 macrophages in HG-treated septic lung tissues and THP-1-derived macrophages under LPS stimulation was accompanied by the activation of NLRP3 inflammasome and NF-κB signaling. HG treatment had an inhibitory effect on the NLRP3 inflammasome, as evidenced by reduced expression of NLRP3 and cleaved caspase-1 and reduced levels of IL-1β and IL-18. IL-1β has been identified as a key cytokine causing acute lung injury [55] [PMID: 11,413,160]. NF-κB, which usually consists of a p50/p65 heterodimer, is isolated in the cytoplasm by binding to IκBα inhibitors in the resting state. Upon stimulation, IκBα is phosphorylated and then dissociated from the NF-κB complex, which liberate NF-κB to the nucleus and activate NF-κB target inflammatory genes. Inhibition of NF-κB pathway has been reported to alleviate ALI [56] [PMID: 34,637,694]. Recent research has reported that HG treatment alleviated ischemia/reperfusion injury by inhibiting the NF-κB pathway [49] [PMID: 32,848,779]. The results showed that, as expected, HG effectively reduced the phosphorylation of NF-κB (p65) and IκBα in ALI rats and LPS-induced THP-1 cells, blocking nuclear translocation of p65. Thus, our study indicated that HG likely exerted anti-inflammatory effects in ALI through inhibiting the polarization of macrophages toward M1 phenotype by suppressing the activation of NLRP3 inflammasome and NF-κB pathways.

The regulatory effect of NF-κB on NLRP3 inflammasome activation has been confirmed in other acute lung injuries induced by carrageenan [57] [PMID: 28,461,340]. However, it has yet to be demonstrated that how NF-κB and NLRP3 signalings interact and impart protection in HG administration. Our study showed that the treatment of HG further emphasized the inhibitory effect of NF-κB inhibitor BAY11-7082 on NLRP3 inflammasome activation, macrophages polarization, and inflammatory cytokine release. Nevertheless, the reduction in these data was not statistically significant, indicating that HG-induced NLRP3 inflammasome inhibition was partially modulated by NF-κB pathway. Other mechanisms may underlie the protective effects of HG in ALI rats, which require an in-depth exploration. Reactive oxygen species (ROS)-mediated NLRP3 inflammasome activation has been reported to play a role in the pathogenesis of septic ALI [58] [PMID: 33,894,359]. The changes of relevant indicators of oxidative stress (SOD, MDA, GSH) in the present study might be associated with increased formation ROS. Therefore, in ALI, HG prevents NLRP3 inflammasome activation by inhibiting ROS may be another possible mechanism. This could be further explored in our future research by ROS scavengers or inhibitors.

## 5 Conclusion

Based on these data, our results demonstrate that HG has superior protective effects in CLP-induced rats by inhibiting inflammation damage, which are largely dependent on the inhibition of macrophage polarization by inhibiting NF-κB-regulated NLRP3 pathway.

## Data Availability

The data that support the findings of this study are available from the corresponding author upon reasonable request.
